# CBLL1 is hypomethylated and correlates with cortical thickness in transgender men before gender affirming hormone treatment

**DOI:** 10.1038/s41598-023-48782-2

**Published:** 2023-12-07

**Authors:** Rosa Fernández, Leire Zubiaurre-Elorza, Andrea Santisteban, Natalia Ojeda, Sarah Collet, Meltem Kiyar, Guy T’Sjoen, Sven C. Mueller, Antonio Guillamon, Eduardo Pásaro

**Affiliations:** 1 Centro Interdisciplinar de Química E Bioloxía - CICA. Departamento de Psicología, Universidade da Coruña, Grupo DICOMOSA, Campus Elviña S/N, 15071 A Coruña, Spain; 2 Instituto de Investigación Biomédica de A Coruña (INIBIC), 15071 Oza, A Coruña, Spain; 3 Departamento de Psicología, Facultad de Ciencias de la Salud, Universidad de Deusto, Bilbao, Spain; 4 Department of Endocrinology, Ghent University Hospital, 9000 Ghent, Belgium; 5 Department of Experimental Clinical and Health Psychology, Ghent University, 9000 Ghent, Belgium; 6 Department of Endocrinology, Center for Sexology and Gender, Ghent University Hospital, 9000 Ghent, Belgium; 7 Departamento de Psicobiología, Facultad de Psicología, Universidad Nacional de Educación a Distancia, 28040 Madrid, Spain

**Keywords:** Epigenetics in the nervous system, Sexual behaviour

## Abstract

Gender identity refers to the consciousness of being a man, a woman or other condition. Although it is generally congruent with the sex assigned at birth, for some people it is not. If the incongruity is distressing, it is defined as gender dysphoria (GD). Here, we measured whole-genome DNA methylation by the Illumina © Infinium Human Methylation 850k array and reported its correlation with cortical thickness (CTh) in 22 transgender men (TM) experiencing GD *versus* 25 cisgender men (CM) and 28 cisgender women (CW). With respect to the methylation analysis, TM *vs.* CW showed significant differences in 35 CpGs, while 2155 CpGs were found when TM *vs.* CM were compared. With respect to correlation analysis, TM showed differences in methylation of *CBLL1* and *DLG1* genes that correlated with global and left hemisphere CTh. Both genes were hypomethylated in TM compared to the cisgender groups. Early onset TM showed a positive correlation between *CBLL1* and several cortical regions in the frontal (left caudal middle frontal), temporal (right inferior temporal, left fusiform) and parietal cortices (left supramarginal and right paracentral). This is the first study relating *CBLL1* methylation with CTh in transgender persons and supports a neurodevelopmental hypothesis of gender identity.

## Introduction

Gender identity is the consciousness of being male, female, or another gender identity. This inner sense may or may not align with a person’s sex as assigned at birth or to a person’s primary or secondary sex characteristics^1^. Transgender persons can fall within the binary male/female system [transgender men or transgender women (TM, TW, respectively)] or as non-binary (genderqueer, gender nonconforming, gender neutral, etc.). Gender identity emerges in the individual because of a constellation of interacting genetic, epigenetic, hormonal, and environmental variables. Some transgender people may experience gender incongruence^2^ and gender dysphoria (GD)^1^ and look for hormonal, and sometimes surgical, gender affirmation treatment.

Since the pioneering work by Swaab^3^ there has been an increase in the exploration of the role of biological variables in gender identity. Structural and functional MRI brain studies show the existence of brain endophenotypes associated to male and female cisgender individuals (CM and CW), people who show congruence between their gender identity and sex assigned at birth , and to transgender persons. Endophenotypes have been reported with respect to cortical thickness (CTh)^4^ and cortical surface area^5^. Moreover, white matter microstructure also shows differences among these four binary groups^6–8^. So, based on the sexual differentiation of the brain, a neurodevelopmental cortical hypothesis was suggested to explain the emergence of these endophenotypes^9^. This hypothesis has found further support by recent functional stationary^10^ and dynamic^11^ fMRI studies, as well as whole-brain dynamic approaches^12^, which also documented brain endophenotypes associated with CM and CW and binary transgender people.

Although cortical surface area is a sensitive measure to distinguish brain endophenotypes between cisgender and transgender populations^5^, CTh measure is less heritable than surface area^13–15^, more dynamic through the lifespan and suitable to search the genetic-environmental interplay, as previously reviewed in clinical and non-clinical populations^16^. Cerebral CTh, which reflects the number of cortical cells, their physiological state, and myelination, depends on age and sex. There is a monotonic thinning of the cortex from early childhood until senescence, but the trajectories of this process are different in different structures^17^ and CTh increases as well as decreases have been reported around puberty^18, 19^. Sex differences in CTh have been reported at all ages^20, 21^. Moreover, the thinning process is related to the efficiency of the androgen receptor^22^. Age- and sex-related changes in CTh suggest a neurodevelopmental process controlled by genetic, epigenetic, and hormonal variables.

The sexual differentiation of the brain depends on genetic and hormonal mechanisms as well as on epigenetic processes. In fact, sex chromosomes, gonadal hormones, and epigenetic mechanisms (DNA and RNA methylation) intertwine to shape brain development and influence behavior in mammals^23^. A growing number of studies demonstrate that there are sex differences in the brain epigenome and that brain sex differentiation requires the interaction between sex hormones and different epigenetic mechanisms^24, 25^.

Genetic studies in transgender people are only recently gaining attraction. For example, such studies have found an association between the four binary gender identities and polymorphisms in androgen and estrogen steroid receptors and enzymes implicated in the sexual differentiation of the brain^26–30^. Moreover, steroid receptor coactivators (SRC1 and SRC2) of estrogen receptors are influential in the masculinization of the brain^31^ and SRC1 and SRC2 polymorphisms were recently associated with GD^32^.

Epigenetic studies disentangle the relationships between genes and the influence of the internal and external environment on gene expression. In mammals DNA methylation, as well as the methylation of RNA messenger (m6A mRNA), play an important role in brain sexual differentiation and behavior^23, 25, 33^. To date, only two studies of whole-genome DNA methylation have been performed in a transgender population, before and during gender affirming hormone treatment (GAHT). These studies demonstrated the existence of characteristic epigenetic marks in the transgender population even before GAHT^34^ as well as the existence of specific epigenetic marks due to GAHT after 6 and 12 months of treatment^35^. Furthermore, methylation analysis of the regulatory region of *ESR1* demonstrated sex differences in the methylation degree and that both TW and TM had significantly lower levels of methylation in this region compared to cisgender men prior to receiving GAHT^36, 37^.

In the present study, we assessed the relationship between DNA methylation and brain CTh in transgender and cisgender people. To this aim, we performed an epigenome-wide association analysis (DNA methylation) and correlated the findings with CTh in transgender men experiencing GD before GAHT and compared these with cisgender men and women. Because of the cortical neurodevelopmental hypothesis^9^, we anticipated that genes related to development would emerge and correlate with brain cortical regions.

## Results

### Demographic data

Data on age, ethnicity and smoking are presented in Table 1.

**Table 1:** Participants’ demographic data.

	Transgender men	Cisgender women	Cisgender men	Statistics (*p* value)
Age (mean ± SD)	29.36 ± 11.50	32.29 ± 8.77	26.36 ± 5.89	K-W = 6.14 (.05)
Ethnicity	White	White	White	
Smoking (never/current/previously)	12/9/1	27/1/0	20/5/0	*X*^*2*^ = *13.75* (.008)
Age of onset (early/late)	12/10			
Subgroups				
Age (mean ± SD)	Early = 30.25 ± 14.77			Mann–Whitney U = 45.00 (.35)
	Late = 28.30 ± 6.27			
Smoking (never/current/previously)	Early = 7/4/1			X^2^ = 1.27 (.53)
	Late = 5/5/0			

### Global methylation analysis

**Figure 1:**
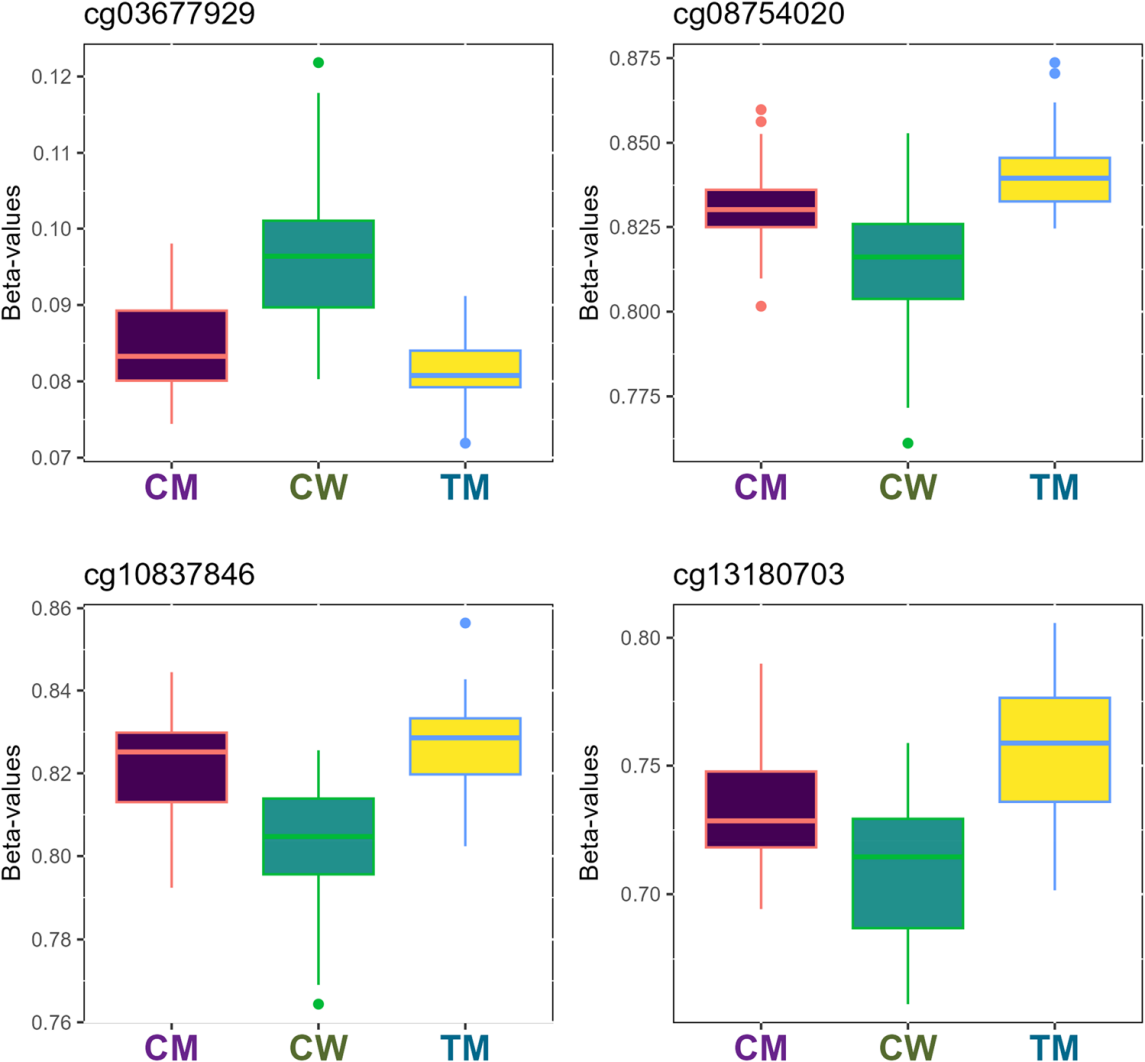
Boxplot representation of the top four most relevant differentially methylated CpG sites in the cisgender women (CW)—transgender men (TM) comparison. Methylation β-values were represented for the top four differentially methylated CpG sites in the CW-TM comparison. Vertical lines in each box represent β-values standard deviation between individuals of the same group, whereas horizontal lines correspond to average β-values of the groups for the probe. Outliers mean individuals whose β-values are extreme in comparison to the average. The samples are coloured according to the variable “group” (purple for cisgender men, green for cisgender women and yellow for transgender men).

**Figure 2:**
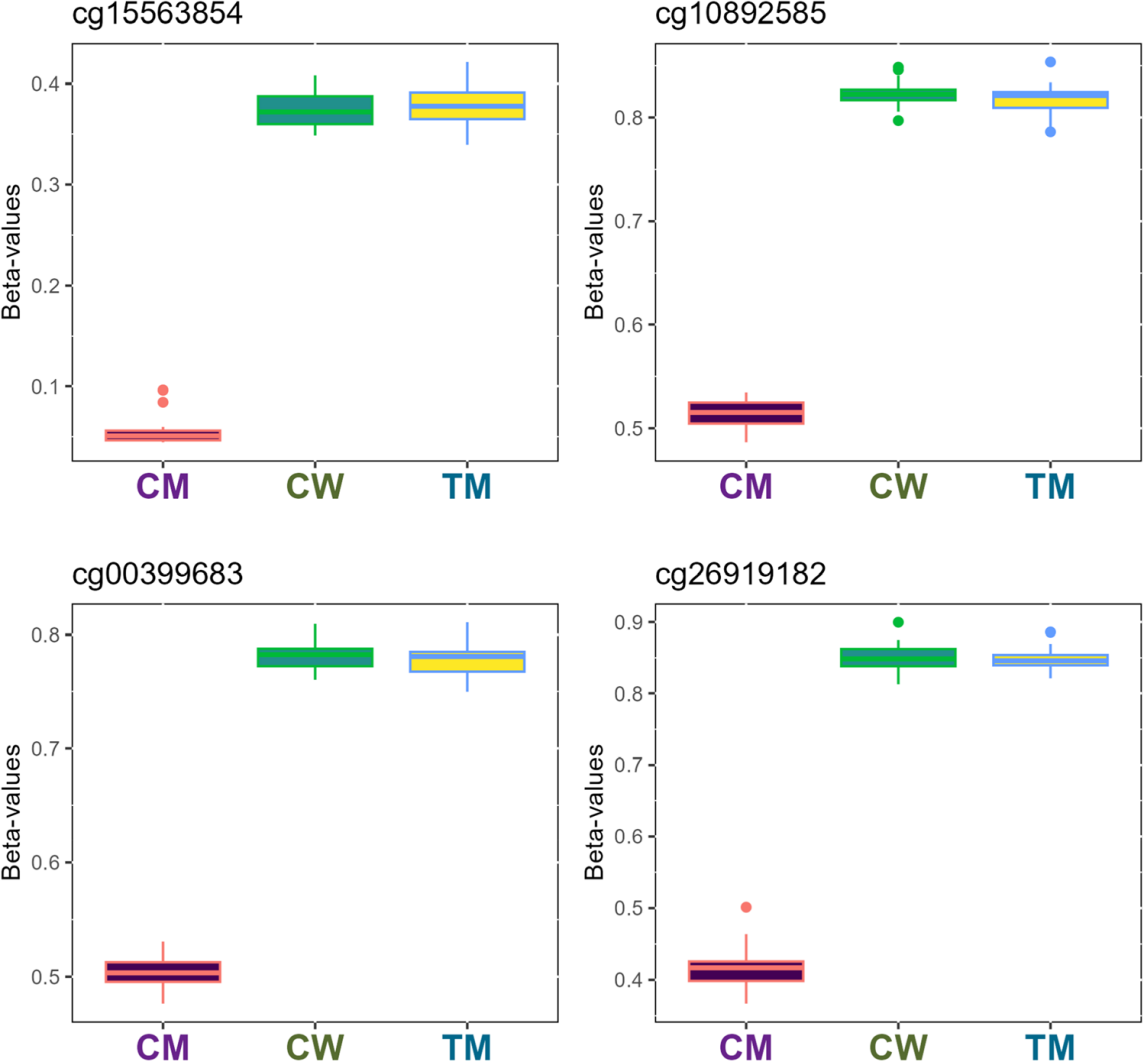
Boxplot representation of the top four most relevant differentially methylated CpG sites in the cisgender men (CM)—transgender men (TM) comparison. Methylation β-values were represented for the top four differentially methylated CpG sites in the CM-TM comparison. Vertical lines in each box represent β-values standard deviation between individuals of the same group, whereas horizontal lines correspond to average β-values of the groups for the probe. Outliers mean individuals whose β-values are extreme in comparison to the average. The samples are coloured according to the variable “group” (purple for cisgender men, green for cisgender women and yellow for transgender men).

**Figure 3:**
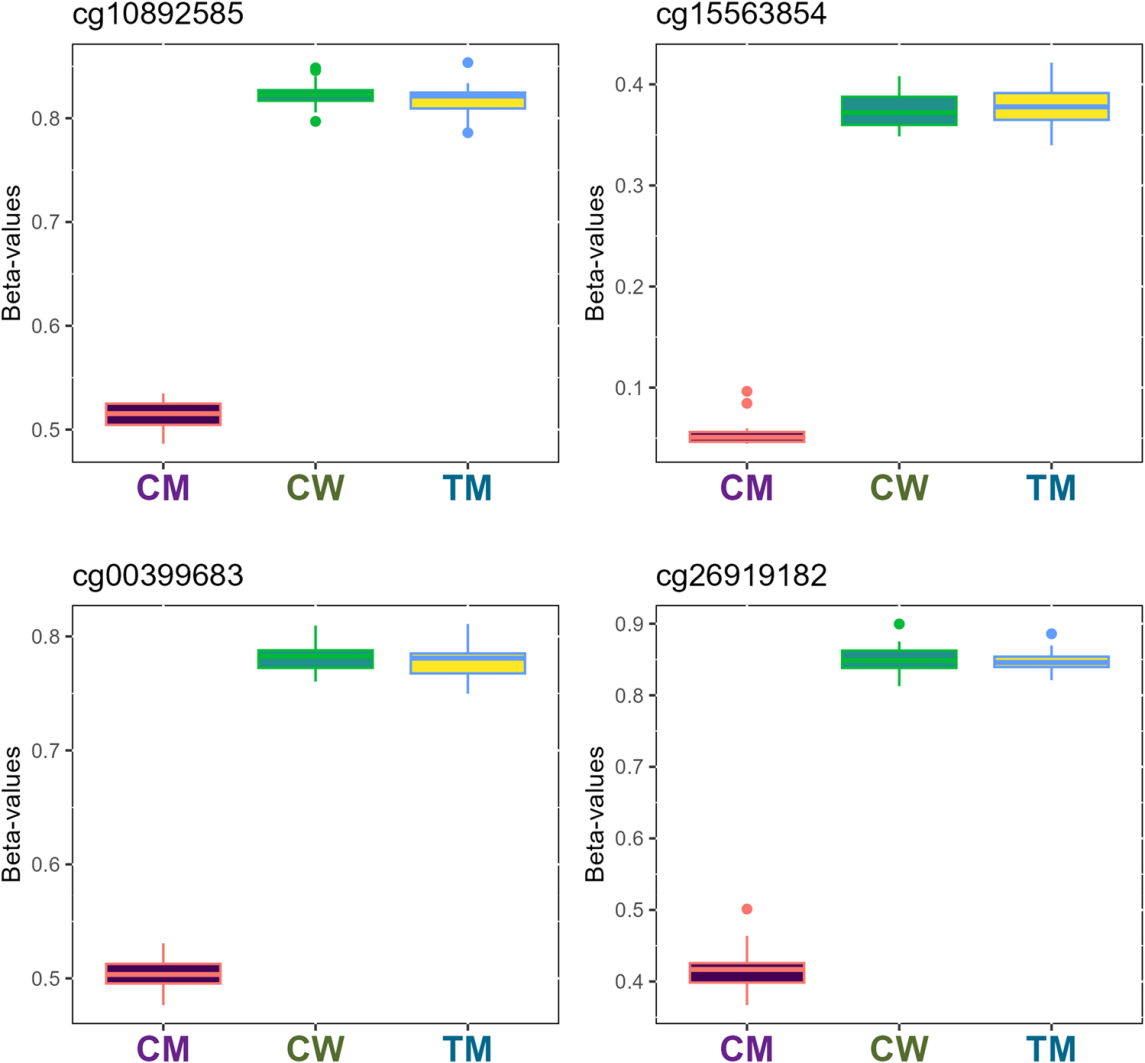
Boxplot representation of the top four most relevant differentially methylated CpG sites in the cisgender men (CM)—cisgender women (CW) comparison. Methylation β-values were represented for the top four differentially methylated CpG sites in the CM-CW comparison. Vertical lines in each box represents β-values standard deviation between individuals of the same group, whereas horizontal lines correspond to average β-values of the groups for the probe. Outliers mean individuals whose β-values are extreme in comparison to the average. The samples are coloured according to the variable “group” (purple for cisgender men, green for cisgender women and yellow for transgender men).

Before correlation analysis, a differential methylation analysis was performed from our 75 individuals (22 TM, 25 CM, and 28 CW) where a pair-wise comparison between individual groups (CW-TM, CM-TM, and CW-CM) was made to obtain significant CpG sites (*p*-value < 0.05). Thus, 35 CpG sites were significant for CW-TM comparison (Fig. 1), 2155 for CM-TM (Fig. 2) and 5684 for CM-CW (Fig. 3) of a total of 813199 CpG sites tested. These genes were involved in numerous biological processes relating to neuroactive ligand-receptor interaction, gap junction, glutamatergic synapse, serotonergic synapse, GABAergic synapse, inflammatory mediator regulation, among others (Supplemental Material Tables S1, S2 and S3).

**Figure 4:**
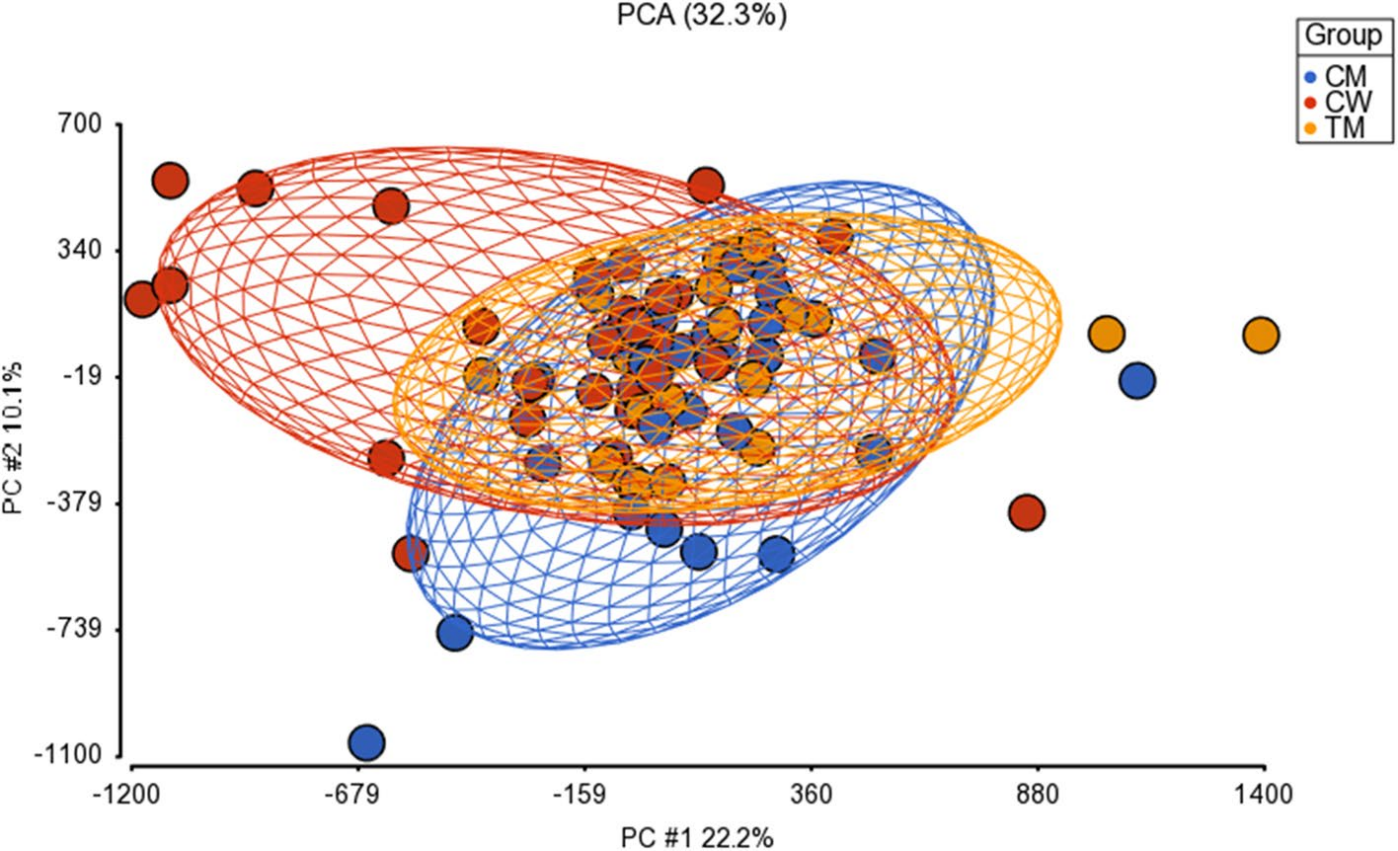
Principal components analysis (PCA) showing methylation profiles of the study samples. Each sample is represented by a dot, the axes are the first two PCs, the percentages indicate the fraction of variance explained by each PC. The number at the top (32.3%) is the variance explained by the first two PCs. The samples are coloured according to the variable “group” (blue for cisgender men, red for cisgender women and yellow for transgender men).

Due to the existence of different significant CpG sites, we included variables of cortical regions in our methylation study to make a correlation analysis between these characteristics and patrons of methylation (Fig. 4).

### MRI data: CTh differences

Regionally statistically significant results emerged in some cortical regions (see *Supplemental *Tables S4 and S5). Cisgender men showed a thicker cortex with respect to CW in bilateral fusiform areas, the left medial orbitofrontal and pars opercularis regions as well as the right middle and transverse temporal regions. However, none of these areas survived Bonferroni correction. Small to medium effect sizes were present in all these analyses.

Regarding early or late onset of GD in TM individuals, defined as an onset of GD that occurs during childhood or pubertal/adult period respectively, regional analyses pointed out the following (see *Supplemental *Tables S6 and S7*)*: (1) CM compared with CW had a thicker cortex in the left medial orbitofrontal and pars opercularis region as well as in the right fusiform area; (2) CM showed higher CTh values in comparison with early onset TM in the left pars opercularis and postcentral areas; (3) late onset when compared to early onset TM had a thicker cortex in the left pars triangularis and postcentral regions and right superior temporal and transverse temporal regions and also in the latter region in comparison with CW. All the above-mentioned results were statistically significant (*p* < 0.05), although none remained significant after Bonferroni correction.

### Methylation & MRI data

**Figure 5:**
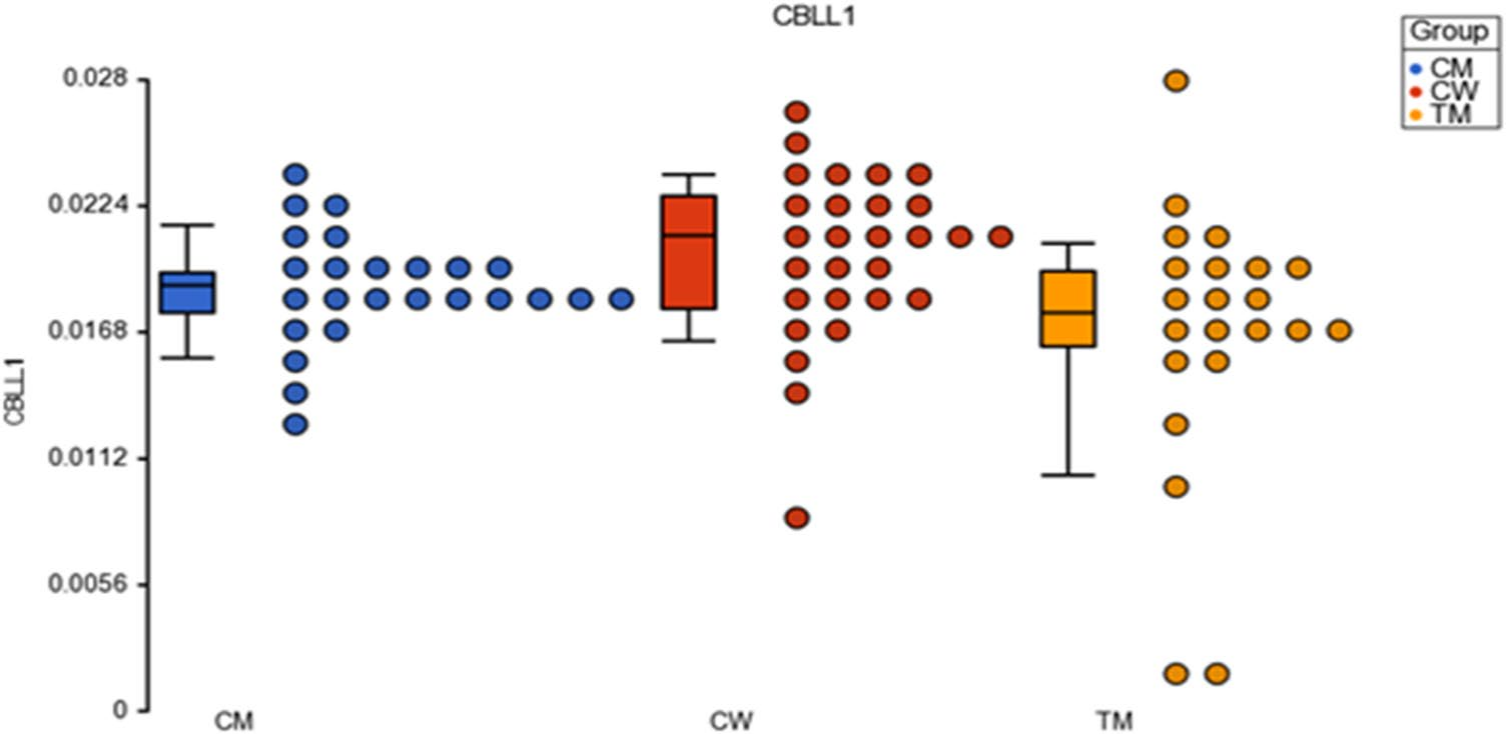
Dotplot showing M-value data for the* CBLL1* gene. Each sample is represented by a dot, which corresponds to the overall degree of methylation (M-value data). The samples are colored according to the levels of the variable “group”: blue for cisgender men, red for cisgender women and yellow for transgender men. The middle line is the median; the box represents the upper and the lower quartile, while the whiskers correspond to the 90th and 10th percentiles of the data.

The ANOVA test comparing methylation by groups and by CTh (CM *vs*. CW, CM *vs*. TM, and CW *vs*. TM) showed statistically significant differences in two genes: *CBLL1* and *DLG1* (Table 2; Fig. 5)*.* According to the Enrichment Score study, these genes are involved in neuronal myelination, *CBLL1* in the CNS and *DLG1* in the peripheral nervous system. Specifically, *CBLL1* is involved in messenger RNA methylation (see Supplemental Material S5).

**Table 2:** Correlation analysis between cortical thickness and *CBLL1* and *DLG1* methylation.

Comparisons	Differences	*p *value	Description
*CBLL1*			
p-value (Group * TOTAL cortical thickness) p < .001			
(CM *vs*. CW)	2.33	< .001	CM up *vs*. CW
(CM *vs*. TM)	23.61	< .001	CM up *vs*. TM
(CW *vs*. TM)	21.29	< .001	CW up *vs*. TM
p-value (Group * Left hemisphere cortical thickness) p < .001			
(CM *vs*. CW)	1.01	.80	CM up *vs*. CW
(CM *vs*. TM)	22.74	< .001	CM up *vs*. TM
(CW *vs*. TM)	21.73	< .001	CW up *vs*. TM
*DLG1*			
p-value (Group * Left hemisphere cortical thickness) p < .001			
(CM *vs*. CW)	-10.74	.003	CM down *vs*. CW
(CM *vs*. TM)	8.63	.01	CM up *vs*. TM
(CW *vs*. TM)	19.37	< .001	CW up *vs*. TM

This table shows the methylation correlation analysis of *CBLL1* and *DLG1* when comparing the groups (TM transmen, CM cismen and CW ciswomen) in total, left and right hemispheres. Only results with statistical significance are shown.

### ANOVA test results: CBLL1

With respect to the *CBLL1* gene, statistically significant differences after FDR correction were found in total CTh and in the left hemisphere, but not in the right hemisphere.

### Total CTh

In the *CBLL1* gene, the methylation degree was higher in cisgender men than in cisgender women (CM > CW) (*p* < 0.001) (Table 2). Similarly, the difference in *CBLL1* methylation was also significantly higher in CM than in TM (CM > TM) (*p* < 0.001) (Table 2).

However, a difference also emerged between CW and TM indicating a higher methylation degree in CW than in TM (CW > TM) (*p* < 0.001) (Table 2). Thus, the *CBLL1* methylation degree followed the sequence CM > CW > TM. The highest methylation degree was found in CM, while TM showed the lowest. Therefore, this gene was hypermethylated in cisgender men and women relative to transgender men, where it was hypomethylated (Table 2).

### Left hemisphere CTh

The pattern in the left hemisphere reflected that of the total CTh, namely, a statistically significant overall effect (*p* < 0.001) (Table 2) following the same sequence: CW > CM > TM. However, while the comparisons between CM *vs*.TM (*p* < 0.001) and CW *vs.* TM (*p* < 0.001) were statistically significant, the differences between the two cisgender groups (CM *vs*. CW; *p* = 0.80) was not (Table 2).

### ANOVA test results: DLG1

#### Left hemisphere CTh

After FDR correction, the *DLG1* gene showed a slightly different pattern. First, statistically significant differences were only found in the left hemisphere. The difference in methylation was statistically significant (*p* = 0.003) when we compared CM to CW, with a higher methylation degree in CW (Table 2). When we compared CM to TM, the difference was also significant (*p* = 0.01), with a higher methylation degree in CM than in TM (Table 2). Finally, the difference was also significant (*p* < 0.001) when we compared CW to TM. The methylation degree was higher in CW than in TM. Therefore, this gene was hypermethylated in the cis population (CM and CW) and hypomethylated in TM, but with higher methylation in CW than CM (CW > CM > TM).

### Correlation between regional CTh and Methylation data

Neither cisgender participants nor late onset TM showed a statistically significant correlation that survived Bonferroni correction (*p *≤ 0.001) between CTh regions and methylation data. The early onset TM group showed a positive association that survived Bonferroni correction (*p* ≤ 0.001) between *CBLL1* methylation and left caudal middle frontal (r = 0.84) fusiform (r = 0.87) and supramarginal (r = 0.89), as well as with the right inferior temporal (r = 0.90) and paracentral regions (r = 0.87). No statistically significant results were found in respect to the* DLG1* gene.

## Discussion

This study is the first, to our knowledge, to demonstrate associations between wide DNA methylation and brain structural measures in transgender people. Two main findings emerged. First, TM showed differences in the methylation degree in two genes, *CBLL1* and *DLG1* that correlated with global and left hemisphere CTh. Both genes were hypomethylated in TM with respect to CW and CM. Second, early onset TM showed a positive correlation between *CBLL1* methylation and several CTh regions. Methylation of *CBLL1* correlated positively with regional CTh in some regions of the frontal (left caudal middle frontal), temporal (right inferior temporal and left fusiform) and parietal cortices (left supramarginal and right paracentral). As was predicted, a gene related with cortical development, *CBLL1*, showed differences in the methylation degree between cisgender and transgender groups.

To study the cortical development of the brain, both volume and CTh values are commonly measured and reported by MRI studies. Regarding the latter, CTh is an extremely dynamic measurement that presents changes across the lifespan^19^. CTh increases in almost all brain areas from the neonatal period to childhood^38^, whereas there is a progressive cortical thinning during adolescence^19^. With respect to GD, a variety of results have been reported in TM before GAHT. On the one hand, some studies have found a thicker cortex in TM with respect to cisgender populations^4, 39^ while a mega-analysis did not find statistically significant differences in TM^5^.

Evaluation of *CBLL1* methylation showed differences between groups in respect to total and left hemisphere CTh, being hypomethylated in TM respect to cisgender groups. There are reports in the literature that relate *CBLL1* with cortical development through complex mechanisms that involve, among others, estrogen receptors. These receptors play a key function in the sexual differentiation of the brain of mammals.

*CBLL1,* which encodes the HAKAI protein, is characterized as an E-cadherin-binding protein that is predominantly expressed in the brain^40^ and has regulatory actions on the alfa estrogen receptor (ERα). It is known that HAKAI modulates ERα transactivation through competitive interference with the coactivators SRC1 and SRC2 by binding to ERα^41^, thereby regulating the expression of ERα target genes**.** Interestingly, in previous studies, we have found that some polymorphisms in the *ESR1* gene, and in SRC1 and SCR2 coactivators, were associated with gender variants^32^.

Moreover, HAKAI have other mechanisms to influence brain development and differentiation through mRNA methylation. This protein is a conserved component of the N6-methyladenosine (m6A) transferase complex for mRNA methylation. This modification plays a central role in almost every aspect of mRNA metabolism and is essential for several biological processes such as myelination, cell differentiation, DNA repair, neurogenesis, sex determination, and silencing the second X chromosome in females^42–44^. Even more, the functioning of the m6A molecular machinery in the nervous system is essential for the life of mammals since m6A marks the "tempo" of corticogenesis^45^ and is also implicated in oligodendrocyte plasticity during adulthood^46^.

The three binary variants also showed differences in *DLG1* methylation in relation to the CTh of the left hemisphere. *DLG1* gene is fully involved in brain development intervening in cellular intimate processes and myelination. Respect to the later, we should underscore that sex differences were reported respect to white matter microstructure in our species^44, 48^.

The *DLG1* gene*,* also called SAP97, is a scaffolding protein that participates in the control of key cellular processes such as polarity, proliferation, and migration as well as in the trafficking of membrane proteins^49, 50^. This gene is widely expressed throughout the brain, concentrating at the postsynaptic space in the cerebral cortex^51^. In addition, SAP97 is widely distributed in excitatory synapses, where it is strongly involved in the trafficking and localization of NMDA and AMPA-type glutamate receptors, regulating synaptic plasticity^52^. Cotter et al.^53^ showed that *Dlg1* acts as an inhibitor of Schwann cell myelination, thus *Dlg1* silencing during mouse SNP myelination generates a hypermyelination phenotype. In contrast, *Dlg1* overexpression reduces myelination^53^. In Schwann cells, Dlg1 interacts with PTEN (phosphatase and tensin homolog deleted on chromosome 10)^54^ to inhibit stimulation of axonal myelination. This mechanism limits the thickness of the myelin sheath and prevents over myelination.

Early and late onset comparison can provide a view of gender identity development and the organizational and activational influences of sex hormones. Puberty marks the early or the late onset GD classification. Early GD emerges before puberty while late GD onset occurs during or after puberty^55, 56^.

The age of onset of GD is a variable of interest unaddressed in MRI studies. However, it was recently reported that age of onset of GD plays a role in the modulation of cerebral metabolite concentrations in TM^57^. In the present study, we found that those TM with early onset GD had a thinner cortex compared to late onset TM and CM. However, these differences did not maintain statistical significance after the Bonferroni correction and so may be due to the small sample size analysed. Nevertheless, early onset TM showed a positive relationship between *CBLL1* methylation and CTh in several cortical regions. A possible explanation is that this gene modulates its activity in early onset GD TM to achieve optimal CTh values in some specific regions of the brain. For example, the supramarginal region, one of the cortical regions in which CTh positively correlates with *CBLL1* methylation only in early onset TM participants, is a crucial region for GD in TM. Resting state functional MRI studies have involved the parietal lobe in own body self-referential processing^58^ and it is a key piece to understand TM stationary and dynamic functional connectivity^10, 11^. As we saw above, *CBLL1* functions are mainly developmental, our findings support two different developmental pathways for early and late GD onset TM and signal the possible implication of specific regions of the cortex in these differences. Our research design precludes to determine if either internal (i.e., hormones), or external (familial/social) variables or their interaction influence *CBLL1* differential methylation at different stages of brain development in early and late onset transgender men.

Some limitations of the current study were the total sample size and the sample size of each subgroup (early *vs.* late onset). The study we have carried out aimed to obtain very homogeneous groups, so some individuals have been eliminated, which has resulted in even smaller groups. A major challenge in the study of DNA methylation is the study of a non-stratified population^59^ that can have a significant impact on the results, as they can lead to false positives or false negatives. In our study, a comprehensive sample selection based on ethnicity and geographic origin was applied, but individuals with drug abuse or associated psychiatric illnesses from the study were removed. This has resulted in a homogenous population, eliminating or reducing the effects of population stratification, but at the same time has resulted in a smaller population. Besides, our results only can apply to binary cisgender heterosexuals and transgender men diagnosed with GD and sexually attracted to women. Moreover, finding an association between DNA methylation and brain structure does not prove that DNA methylation markers imply a cause or consequence of brain findings or are due to other confounding variables^16^.

However, the main strength of our work is that *CBLL1* and *DLG1* have been identified as candidate genes in TM due to its relationship with CTh. Moreover, early, and late onset TM were epigenetically and cortically differentiated. These robust differences remain after rigorous standards were applied with a very conservative statistical correction (i.e. Bonferroni and FDR corrections).

## Concluding remarks

What do we know with respect to the variables that could intervene in brain development of binary cis and transgender persons. At the genetic level we have shown that either the alfa or the beta estrogen receptor polymorphisms^28, 29^ as well as SCR1 and SCR2 polymorphisms are related to TM^32^. Moreover, epigenetic analyses show that methylation of the promoter region of the alpha estrogen receptor is also associated with TM^37^. It seems, at least in our studies, that any genetic or epigenetic changes that could affect normative functioning of transcription by estrogens is associated with TM. The present findings with respect to *CBLL1* support SCR1 and SCR2 implication in TM and envision the possible participation of mechanisms implicating mRNA methylation in the regulation of the thickness of the cortex in our species.

Finally, our findings suggest that *CBLL1* and *DLG1* may participate in the modulation of CTh in humans and they distinguish three cortical endophenotypes related to gender identity (i.e., CM, CF, TM). Moreover, they also suggest epigenetic differences related to the cortex between early and late GD onset TM. All these results support a cortical hypothesis that suggests that different rates of development, in specific cortical regions, could underlie gender identity and its variants ^9, 38^. Future studies should include larger and homogeneous transgender samples with different onsets of GD. All these findings support a neurodevelopmental hypothesis^9^ to explain the development of the binary male and female gender identities and point out they have a biological counterpart.

## Methods

### Participants’ characteristics

One hundred and twelve individuals, belonging to a larger study, participated in the current investigation. For this study, the inclusion criteria for transgender people were: (1) being aged 17 years or older; (2) the presence of GD according to DSM-5; (3) identification with the gender other than the one assigned at birth (male or female), and (4) having no prior history of hormonal treatment. The exclusion criteria for cisgender and transgender populations were: (a) current or past drug consumption; (b) the presence of psychiatric, neurological and/or hormonal disorders, or a major medical condition as assessed by anamnesis and (c) history of cranial contusion or injury. In addition, the Mini-International Neuropsychiatric Interview^60^ was carried out by a clinical psychologist, assessing possible undiagnosed psychiatric symptoms in participants. During their first clinic visit to enrol for GAHT, self-reported sexual orientation was solicited and only people attracted to the opposite gender relative to their gender identity were included in the study. TM also self-reported the age of GD onset^55^, the stage of life they have experienced gender incongruent feelings (i.e., childhood, pubertal age, and adult life). Early onset GD corresponds to the childhood period and late onset GD to pubertal or adult life^55^.

From the initial 112 participants 37 were excluded after the inclusion/exclusion criteria were applied for the following reasons: 32 individuals did not have an MRI study; 4 reported drug consumption and 1 had no sociodemographic data. Therefore, 22 TM (mean age: 29.32 ± 11.52) before GAHT and 53 cisgender individuals [25 CM (mean age: 26.52 ± 5.86) and 28 CW (mean age: 31.61 ± 9.08)] were recruited at the Center for Sexology and Gender, Department of Endocrinology at Ghent University Hospital (Belgium). Cisgender individuals were enrolled via media advertising and by word of mouth. All participants in the study received 20 Euros as compensation. Written informed consent was obtained from all participants after a full explanation of the procedures. The study was approved by the ethical committee of Ghent University Hospital (Belgium) and it was performed in accordance with relevant regulations and guidelines.

### DNA methylation analysis

Genomic DNAs were extracted from peripheral blood using the DNeasy Blood & Tissue Kit (Qiagen), and an aliquot of 1 µg DNA per subject was processed for bisulphite conversion (Zymo Research EZ Methylation Kit), according to the manufacturer’s instructions. Subsequently, DNA methylome was analysed using the Illumina © Infinium Human Methylation 850k BeadChip array (Illumina, San Diego, CA, USA) that assesses 862,927 cytosine–phosphate–guanine (CpG) sites throughout the genome, covering 99% of RefSeq genes, 95% of CpG islands and offers high coverage of enhancer regions. Studies have shown a correlation between DNA methylation in blood and brain tissue^61^. The array was scanned with the Illumina iScan SQ system and the image intensities were extracted with Genome Studio (2011.1). DNA quality controls, data normalization and statistical filters were performed with the Partek® Genomics Suite® v7.19.1018. Methylation of the X and Y chromosomes were excluded from the analysis in order to compare XX and XY populations. Functional normalization, NOOB background correction, and dye correction were applied.

All analyses were done by the Partek® Genomics Suite® software, version 7.0 and are supported by RStudio. The human reference genome (GRCh37/hg19 assembly) was used to determine the location and features of the gene region using the UCSC Genome Browser^62^.

### MRI acquisition and analysis

The three-dimensional MRI data sets were acquired at Ghent University Hospital (Belgium). High-resolution T1-weighted images were acquired for all participants on a 3-Tesla Siemens Prisma Fit MRI scanner (Siemens Healthcare, Erlangen, Germany) with a 64-channel head coil. The following parameters were used: a MPRAGE sequence with TR/TE = 2300/2.96 ms; TI = 900 ms; 192 slices; flip angle 9 degrees; 256 × 256 matrix and 1 mm^3^ isotropic voxel.

Automated cortical reconstruction of the T1-weighted images was performed using the FreeSurfer (version 6.0.0) image analysis suite (http://surfer.nmr.mgh.harvard.edu). This method was used to create a cortical surface three-dimensional model of CTh using intensity and continuity information previously described in detail^63^. Processing of T1 high resolution images includes several procedures: motion correction, removal of non-brain tissue using a hybrid watershed/surface deformation procedure^64^, automated Talairach transformation, intensity normalization^65^, tessellation of the gray matter/white matter boundary, automated topology correction^66, 67^, and surface deformation to detect gray matter/white matter and gray matter/cerebrospinal fluid boundaries where the greatest shift in intensity defines the transition to the other tissue class^63^. Moreover, the cerebral cortex was divided into different regions according to gyral and sulcal structure information^68^. The resulting representation of CTh is calculated as the distance between tissue boundaries (gray matter/white matter and gray matter/cerebrospinal fluid^63^. All surface models in our study were visually inspected for accuracy.

### Analysis procedure

Firstly, after a quality control and filtering, a wise-pair differential methylation study was performed to compare our groups (CW-TM, CM-TM and CW-CM) to ensure there are significant CpG sites before continuing the analysis. In second place, CTh values (total, left and right hemisphere) were introduced as new variables to apply an ANOVA test to obtain genes which were specially related to CTh and TM. These candidate genes were used to perform a correlation analysis with regional CTh values based on the Desikan atlas^68^. Complementary correlations were performed to investigate the effect of age of onset of GD in the relationship between methylation data and regional CTh. More detailed information is described in the following paragraphs.

### Statistical analysis

#### Sociodemographic data (age, smoking, drugs, race, onset)

Age was tested for normality and homogeneity. Age differences between the three groups were tested using a Kruskal–Wallis test. Categorical variables such as smoking and age of onset were compared using a *X*^*2*^ test of independence. When complementary analyses were performed in a subset of participants (early *vs*. late onset transgender men), the age variable was compared using Mann–Whitney U statistics. All statistical analyses were performed using IBM SPSS Statistics v. 29.

#### Methylation data and functional and regulatory enrichment analysis

To detect the differential methylation in CpG sites that varies across all groups, we compared CW-TM, CM-TM, CW-CM. Then, we performed an ANOVA test comparing groups by CTh: CW-TM, CM-TM, CW-CM.

For each contrast, a *p*-value, Beta difference (Δβ), and M difference (ΔM) were generated. P values were calculated using false discovery rate correction (FDR, *p* < 0.05) and fold change ≥  ± 2 was applied. The distribution of significant CpG sites was examined across functional and regulatory annotations. The GO enrichment analysis was carried out with the Partek® Pathway program (Supplemental Material Table S8).

#### MRI data: regional CTh values

Freesurfer‐generated surfaces were used to calculate CTh estimates according to the 68 regions contemplated in the Desikan atlas^66^. Regional CTh in the above-mentioned areas (34 areas per hemisphere) were compared between the three groups of interest (TM, CW and CM) using a multivariate analysis of covariance. Complementary analyses were performed in order to study the role of age of onset of GD. The reason for this complementary analysis is a previous report where we found metabolomic differences within the TM group regarding early *vs*. late onset of GD^57^. Bonferroni’s post-hoc test was employed to assess CTh differences between groups. Partial squared eta was employed to calculate the effect sizes; around 0.01 is a small size effect, 0.06 is medium, and above 0.14 is large.

#### Methylation correlation with MRI data

Regional CTh correlations with methylation data were conducted, specifically with those genes that showed a different methylation degree between groups. As a secondary analysis, the relationship between CTh and methylation degree was explored considering age at GD onset (early *vs*. late onset). Partial correlations between CTh and genetic data were also performed in early *vs*. late TM. The Smoking variable was introduced as a covariate in all analyses. *MRI data: regional CTh values* and *Methylation and MRI data* sections’ analysis were performed by means of IBM SPSS Statistics v. 29.

#### Multiple comparisons correction

To be statistically conservative, we applied a multi-layered approach of correcting for multiple comparisons at several levels. First, to correct for multiple genes, we used the common FDR correction (*p* < 0.05). Second, to correct for brain regions, we used Bonferroni correction; significance was set at *p* ≤ 0.001 (CTh areas (left/right) *p* = 0.05/34 = 0.001).

### Ethical approval

This study was reviewed and approved by the Ethical Committees of Gent University Hospital, Belgium. The participants provided their written informed consent to participate in this study.

### Supplementary Information

Supplementary Information.

## Data Availability

The datasets presented in this study can be found in GEO online repository. The names of the repository and accession number(s) can be found below: GSE237955. The samples were randomised across EPIC slides.
