# Supplementary material for: CBLL1 is hypomethylated and correlates with cortical thickness in transgender men before gender affirming hormone treatment
Source: Sci Rep. 2023 Dec 7;13:21609. doi: 10.1038/s41598-023-48782-2 (PMC10703770; doi:10.1038/s41598-023-48782-2)
Supplement: Supplementary file 1 — Supplementary Information. [file 41598_2023_48782_MOESM1_ESM.pdf]

Supplemental material

Table S1.

Pathways related with the top four most relevant differentially methylated CpGs in the cisgender women-transgender men (CW-TM) comparison.

Description	N	DE	P.DE	FDR	SigGenesInSet
Chemokine signaling pathway	189	2	0.046	1	GSK3B,PARD3
Wnt signaling pathway	165	2	0.048	1	GSK3B,CCDC88C
Hippo signaling pathway	155	2	0.043	1	GSK3B,PARD3
Insulin signaling pathway	131	2	0.027	1	RHOQ,GSK3B
Alcoholic liver disease	139	2	0.019	1	CAMKK2,GSK3B

Abbreviations: N: number of pathway genes. DE: number of genes that are differentially methylated. P.DE: p-value of the pathway representation. FDR: false discovery rate. SigGenesInSet: significantly differentially methylated genes of the pathway.

Table S2.

Pathways related with the top four most relevant differentially methylated CpGs in the cisgender men-transgender men (CM-TM) comparison.

Description	N	DE	P.DE	FDR	SigGenesInSet
Arginine biosynthesis	20	5	0.005	0.48	GLS2,GLUD1,GOT2,ASS1,NOS1
Phospholipase D signaling pathway	144	18	0.02	0.56	GNA13,ADCY8,ADCY9,DNM1,EGFR,PLCB1,GNA12,GRB2,GRM5,GRM8,PDGFB,PIK3CD,PLCG1,SHC3,PRKCA,PTGFR,SHC1,DGKI,CYTH2
Neuroactive ligand-receptor interaction	346	27	0.022	0.56	ADORA3,GABRA4,GABRB3,GHR,GIPR,MLNR,GRID1,GRIK2,GRIN1,NR3C1,GRM5,GRM8,HTR7,P2RY6,GAL,PDYN,S1PR5,POMC,PARD3,CHRNA10,SLURP1,PTGER1,PTGFR,THRA,TRPV1,CALCR,CCK
Gap junction	87	14	0.002	0.48	TUBB3,ADCY8,ADCY9,CSNK1D,EGFR,TUBB,PLCB1,GRB2,GRM5,TUBB8,PDGFB,PRKCA,PRKCB,PRKG1,SRC
Circadian entrainment	96	15	0.004	0.48	GNB5,ADCY8,ADCY9,PLCB1,GNG4,GRIN1,NOS1,PRKCA,PRKCB,PRKG1,CACNA1C,CALM1,PER3,PER2,CACNA1H
Long-term potentiation	64	9	0.042	0.78	ADCY8,PLCB1,GRIN1,GRM5,PRKCA,PRKCB,RPS6KA2,CACNA1C,CALM1

Glutamatergic synapse	114	16	0.01	0.51	GNB5,ADCY8,ADCY9,SHANK2,PLCB1,GLS2,GNG4,GRIK2,GRIN1,GRM5,GRM8,PRKCA,PRKCB,SLC1A3,CACNA1C,SHANK3
Serotonergic synapse	108	12	0.041	0.78	GNB5,PLCB1,ALOX12B,ALOX15B,GABRB3,GNG4,HTR7,APP,KCND2,PRKCA,PRKCB,CACNA1B,CACNA1C
GABAergic synapse	84	12	0.017	0.56	GNB5,ADCY8,ADCY9,GABRA4,GABRB3,GAD2,GLS2,GNG4,PRKCA,PRKCB,SRC,CACNA1B,CACNA1C
Inflammatory mediator regulation of TRP channels	97	13	0.021	0.56	ADCY8,ADCY9,PLCB1,ASIC1,NTRK1,PIK3CD,PLCG1,ASIC4,PRKCA,PRKCB,MAPK13,SRC,TRPV1,CALM1
Aldosterone synthesis and secretion	95	13	0.02	0.56	CREB3,ADCY8,ADCY9,CYP11A1,PLCB1,PRKD2,ATP1A3,POMC,PRKCA,PRKCB,CACNA1C,CALM1,ORAI1,CACNA1H
Relaxin signaling pathway	126	16	0.01	0.51	CREB3,GNB5,ADCY8,ADCY9,EGFR,PLCB1,GNG4,GRB2,SMAD2,ARRB2,NOS1,PIK3CD,SHC3,PRKCA,MAPK13,SHC1,SRC
Cortisol synthesis and secretion	63	11	0.005	0.48	CREB3,ADCY8,ADCY9,CYP11A1,PLCB1,PBX1,POMC,NCEH1,SP1,CACNA1C,ORAI1,CACNA1H
Growth hormone synthesis, secretion and action	118	14	0.042	0.78	CREB3,ADCY8,ADCY9,PLCB1,GHR,GRB2,PIK3CD,PLCG1,SHC3,PRKCA,PRKCB,MAPK13,SHC1,CACNA1C,BCAR1

Huntington disease	287	27	0.008	0.51	TUBB3,CREB3,DNAH14,COX6B1,AP2B1,TUBB,PLCB1,GPX1,GRIN1,GRM5,HTT,TUBB8,KIF5C,NDUFA13,ATP5MC2,ATP5PF,PPARG,PSMA1,PSMA6,PSMD2,PSMD11,SDHA,SDHD,SLC1A3,SOD1,SP1,UQCRB,VDAC1,CACNA1B,ULK1
Spinocerebellar ataxia	139	17	0.012	0.51	PLCB1,GRIN1,PDYN,PIK3CD,PRKCA,PRKCB,PSMA1,PSMA6,PSMD2,PSMD11,RORA,ATXN1,ATXN2,ATXN8OS,SP1,VDAC1,ULK,PUM1
Prion disease	252	23	0.012	0.51	TUBB3,CREB3,STIP1,COX6B1,TUBB,GRIN1,HSPA1L,HSPA8,TUBB8,KIF5C,NDUFA13,ATP5MC2,ATP5PF,PIK3CD,MAPK13,PSMA1,PSMA6,PSMD2,PSMD11,SDHA,SDHD,SOD1,UQCRB,VDAC1,CACNA1B,CACNA1C
Human cytomegalovirus infection	223	21	0.041	0.78	CREB3,GNA13,GNB5,ADCY8,ADCY9,EGFR,EIF4EBP1,PLCB1,GNA12,GNG4,GRB2,PIK3CD,PRKCA,PRKCB,MAPK13,PTGER1,CX3CL1,SP1,SRC,CALM1,TRADD,BCAR1

Abbreviations: N: number of pathway genes. DE: number of genes that are differentially methylated. P.DE: p-value of the pathway representation. FDR: false discovery rate. SigGenesInSet: significantly differentially methylated genes of the pathway.

Table S3.

Pathways related with the top four most relevant differentially methylated CpGs in the cisgender men-cisgender women (CM-CW) comparison.

Description	N	DE	P.DE	FDR	SigGenesInSet
PPAR signaling pathway	72	17	0.031	1	GLS2,GLUD1,GOT2,ASS1,NOS1
Rap1 signaling pathway	207	50	0.042	1	GNA13,ADCY8,ADCY9,DNM1,EGFR,PLCB1,GNA12,GRB2,GRM5,GRM8,PDGFB,PIK3CD,PLCG1,SHC3,PRKCA,PTGFR,SHC1,DGKI,CYTH2
mTOR signaling pathway	152	37	0.024	1	ADORA3,GABRA4,GABRB3,GHR,GIPR,MLNR,GRID1,GRIK2,GRIN1,NR3C1,GRM5,GRM8,HTR7,P2RY6,GAL,PDYN,S1PR5,POMC,PARD3,CHRNA10,SLURP1,PTGER1,PTGFR,THRA,TRPV1,CALCR,CCK
Longevity regulating pathway - multiple species	60	18	0.023	1	TUBB3,ADCY8,ADCY9,CSNK1D,EGFR,TUBB,PLCB1,GRB2,GRM5,TUBB8,PDGFB,PRKCA,PRKCB,PRKG1,SRC
Glutamatergic synapse	114	33	0.015	1	GNB5,ADCY8,ADCY9,PLCB1,GNG4,GRIN1,NOS1,PRKCA,PRKCB,PRKG1,CACNA1C,CALM1,PER3,PER2,CACNA1H
Insulin secretion	83	23	0.043	1	ADCY8,PLCB1,GRIN1,GRM5,PRKCA,PRKCB,RPS6KA2,CACNA1C,CALM1

Melanogenesis	101	26	0.036	1	GNB5,ADCY8,ADCY9,SHANK2,PLCB1,GLS2,GNG4,GRIK2,GRIN1,GRM5,GRM8,PRKCA,PRKCB,SLC1A3,CACNA1C,SHANK3
Maturity onset diabetes of the young	26	8	0.026	1	GNB5,PLCB1,ALOX12B,ALOX15B,GABRB3,GNG4,HTR7,APP,KCND2,PRKCA,PRKCB,CACNA1B,CACNA1C
Spinocerebellar ataxia	139	36	0.01	1	GNB5,ADCY8,ADCY9,GABRA4,GABRB3,GAD2,GLS2,GNG4,PRKCA,PRKCB,SRC,CACNA1B,CACNA1C
Nicotine addiction	36	12	0.022	1	ADCY8,ADCY9,PLCB1,ASIC1,NTRK1,PIK3CD,PLCG1,ASIC4,PRKCA,PRKCB,MAPK13,SRC,TRPV1,CALM1
Endometrial cancer	56	18	0.025	1	CREB3,ADCY8,ADCY9,CYP11A1,PLCB1,PRKD2,ATP1A3,POMC,PRKCA,PRKCB,CACNA1C,CALM1,ORAI1,CACNA1H
Glioma	74	21	0.04	1	CREB3,GNB5,ADCY8,ADCY9,EGFR,PLCB1,GNG4,GRB2,SMAD2,ARRB2,NOS1,PIK3CD,SHC3,PRKCA,MAPK13,SHC1,SRC
Basal cell carcinoma	63	17	0.041	1	CREB3,ADCY8,ADCY9,CYP11A1,PLCB1,PBX1,POMC,NCEH1,SP1,CACNA1C,ORAI1,CACNA1H
Gastric cancer	147	37	0.019	1	CREB3,ADCY8,ADCY9,PLCB1,GHR,GRB2,PIK3CD,PLCG1,SHC3,PRKCA,PRKCB,MAPK13,SHC1,CACNA1C,BCAR1

Arrhythmogenic right ventricular cardiomyopathy	74	22	0.037	1	TUBB3,CREB3,DNAH14,COX6B1,AP2B1,TUBB,PLCB1,GPX1,GRIN1,GRM5,HTT,TUBB8,KIF5C,NDUFA13,ATP5MC2,ATP5PF,PPARG,PSMA1,PSMA6,PSMD2,PSMD11,SDHA,SDHD,SLC1A3,SOD1,SP1,UQCRB,VDAC1,CACNA1B,ULK1

Abbreviations: N: number of pathway genes. DE: number of genes that are differentially methylated. P.DE: p-value of the pathway representation. FDR: false discovery rate. SigGenesInSet: significantly differentially methylated genes of the pathway.

Table S4.

Quantitative regional estimates for left hemisphere cortical thickness

	Transgender men n=22 mean ± SD	Cisgender women n=28 mean ± SD	Cisgender men n=25 mean ± SD	F-Snedecor Statistic (p)	ηp^2
Bankssts	2.57 ± 0.15	2.57 ± 0.17	2.59 ± 0.12	0.10 (0.91)	0.03
Caudal anterior cingulate	2.63 ± 0.19	2.71 ± 0.20	2.71 ± 0.21	1.10 (0.35)	0.03
Caudal middle frontal	2.55 ± 0.15	2.55 ± 0.12	2.55 ± 0.13	0.01 (0.99)	<0.01
Cuneus	1.99 ± 0.14	1.97 ± 0.12	2.01 ± 0.11	0.84 (0.44)	0.02
Entorhinal	3.43 ± 0.31	3.34 ± 0.23	3.52 ± 0.30	2.67 (0.08)	0.07
**Fusiform**	2.85 ± 0.13	**2.84 ± 0.09**	**2.91 ± 0.08**	**3.54 (0.03)**	**0.09**
Inferior parietal	2.52 ± 0.11	2.52 ± 0.10	2.54 ± 0.11	0.26 (0.77)	<0.01
Inferior temporal	2.90 ± 0.16	2.85 ± 0.14	2.92 ± 0.14	1.33 (0.27)	0.04

Isthmus cingulate	2.40 ± 0.19	2.45 ± 0.17	2.44 ± 0.14	0.48 (0.62)	0.01
Lateral occipital	2.31± 0.12	2.27 ± 0.11	2.33 ± 0.10	1.67 (0.20)	0.05
Lateral orbitofrontal	2.66 ± 0.14	2.64 ± 0.16	2.71 ± 0.13	1.28 (0.29)	0.04
Lingual	2.10 ± 0.13	2.11 ± 0.11	2.17 ± 0.08	2.88 (0.06)	0.08
**Medial orbitofrontal**	2.47 ± 0.15	**2.42 ± 0.11**	**2.53 ± 0.14**	**4.81 (0.01)**	**0.12**
Middle temporal	2.97 ± 0.16	2.91 ± 0.11	2.99 ± 0.14	2.10 (0.13)	0.06
Parahippocampal	2.89 ± 0.25	2.86 ± 0.28	2.95 ± 0.25	0.93 (0.40)	0.03
Paracentral	2.48 ± 0.12	2.50 ± 0.13	2.51 ± 0.15	0.20 (0.84)	<0.01
**Parsopercularis**	2.64 ± 0.16	**2.62 ± 0.12**	**2.74 ± 0.11**	**5.57 (<0.01)**	**0.14**
Parsorbitalis	2.72 ± 0.23	2.75 ± 0.19	2.73 ± 0.22	0.10 (0.90)	<0.01
Parstriangularis	2.52 ± 0.16	2.50 ± 0.10	2.57 ± 0.15	2.00 (0.14)	0.05
Pericalcarine	1.75 ± 0.16	1.70 ± 0.14	1.72 ± 0.11	0.73 (0.49)	0.02

Postcentral	2.15 ± 0.11	2.18 ± 0.11	2.21 ± 0.10	1.22 (0.30)	0.03
Posterior cingulate	2.50 ± 0.17	2.48 ± 0.14	2.53 ± 0.16	0.64 (0.53)	0.02
Precentral	2.64 ± 0.16	2.69 ± 0.12	2.69 ± 0.12	0.80 (0.45)	0.02
Precuneus	2.49 ± 0.11	2.49 ± 0.10	2.53 ± 0.11	1.13 (0.33)	0.03
Rostral anterior cingulate	2.90 ± 0.20	2.87 ± 0.23	2.93 ± 0.20	0.64 (0.53)	0.02
Rostral middle frontal	2.37 ± 0.14	2.35 ± 0.11	2.43 ± 0.13	2.36 (0.10)	0.06
Superior frontal	2.70 ± 0.18	2.66 ± 0.13	2.73 ± 0.14	1.26 (0.29)	0.03
Superior parietal	2.27 ± 0.11	2.28 ± 0.08	2.28 ± 0.11	0.04 (0.97)	<0.01
Superior temporal	2.92 ± 0.19	2.91 ± 0.13	2.97 ± 0.13	1.10 (0.34)	0.03
Supramarginal	2.64 ± 0.14	2.63 ± 0.13	2.65 ± 0.13	0.18 (0.84)	<0.01
Frontal pole	2.76 ± 0.24	2.68 ± 0.22	2.78 ± 0.22	1.38 (0.26)	0.04
Temporal pole	3.62 ± 0.36	3.51 ± 0.33	3.54 ± 0.40	0.46 (0.63)	0.01

Transverse temporal	2.54 ± 0.22	2.56 ± 0.21	2.60 ± 0.25	0.38 (0.68)	0.01
Insula	3.03 ± 0.24	3.02 ± 0.18	3.07 ± 0.16	0.53 (0.59)	0.02

Abbreviations: ηp^2: partial eta squared

Notes: All analyses were covariated by smoking variable and marginal means are reported. Numbers in bold represent areas, comparing groups where statistically significant results were found.

Table S5

Quantitative regional estimates for right hemisphere cortical thickness

	Transgender men n=22 mean ± SD	Cisgender women n=28 mean ± SD	Cisgender men n=25 mean ± SD	F-Snedecor Statistic (p)	ηp^2
Bankssts	2.71 ± 0.17	2.71 ± 0.14	2.73 ± 0.18	0.19 (0.83)	<0.01
Caudal anterior cingulate	2.53 ± 0.18	2.46 ± 0.17	2.52 ± 0.17	1.10 (0.34)	0.03
Caudal middle frontal	2.47 ± 0.17	2.46 ± 0.11	2.44 ± 0.13	0.28 (0.76)	<0.01
Cuneus	2.04 ± 0.11	2.03 ± 0.13	2.09 ± 0.14	1.44 (0.24)	0.04
Entorhinal	3.63 ± 0.27	3.49 ± 0.32	3.62 ± 0.29	1.69 (0.19)	0.05
**Fusiform**	2.88 ± 0.11	**2.86 ± 0.09**	**2.94 ± 0.10**	**4.71 (0.01)**	**0.12**
Inferior parietal	2.62 ± 0.13	2.60 ± 0.11	2.63 ± 0.10	0.40 (0.67)	0.01
Inferior temporal	2.89 ± 0.17	2.85 ± 0.12	2.93 ± 0.13	1.98 (0.15)	0.05

Isthmus cingulate	2.54 ± 0.25	2.44 ± 0.16	2.49 ± 0.18	1.48 (0.23)	0.04
Lateral occipital	2.36 ± 0.13	2.37 ± 0.10	2.42 ± 0.11	1.59 (0.21)	0.04
Lateral orbitofrontal	2.56 ± 0.10	2.59 ± 0.11	2.56 ± 0.12	0.48 (0.62)	0.01
Lingual	2.17 ± 0.14	2.15 ± 0.10	2.21 ± 0.09	1.84 (0.17)	0.05
Medial orbitofrontal	2.39 ± 0.15	2.38 ± 0.14	2.46 ± 0.14	2.78 (0.07)	0.07
**Middle temporal**	3.00 ± 0.16	**2.94 ± 0.14**	**3.04 ± 0.11**	**3.26 (0.04)**	**0.08**
Parahippocampal	2.90 ± 0.21	2.89 ± 0.21	2.95 ± 0.24	0.53 (0.59)	0.02
Paracentral	2.53 ± 0.13	2.51 ± 0.12	2.53 ± 0.14	0.90 (0.91)	<0.01
Parsopercularis	2.56 ± 0.17	2.55 ± 0.15	2.60 ± 0.12	1.04 (0.36)	0.03
Parsorbitalis	2.63 ± 0.17	2.68 ± 0.18	2.60 ± 0.17	1.52 (0.23)	0.04
Parstriangularis	2.43 ± 0.18	2.40 ± 0.14	2.42 ± 0.16	0.21 (0.81)	<0.01
Pericalcarine	1.75 ± 0.16	1.70 ± 0.15	1.70 ± 0.14	0.64 (0.53)	0.02

Postcentral	2.11 ± 0.11	2.18 ± 0.12	2.16 ± 0.11	1.65 (0.20)	0.04
Posterior cingulate	2.54 ± 0.14	2.45 ± 0.15	2.50 ± 0.14	1.84 (0.17)	0.05
Precentral	2.61 ± 0.15	2.58 ± 0.14	2.59 ± 0.12	0.22 (0.80)	<0.01
Precuneus	2.51 ± 0.12	2.50 ± 0.10	2.53 ± 0.10	0.49 (0.62)	0.01
Rostral anterior cingulate	2.80 ± 0.20	2.81 ± 0.16	2.87 ± 0.16	1.08 (0.35)	0.03
Rostral middle frontal	2.23 ± 0.14	2.22 ± 0.09	2.23 ± 0.10	0.04 (0.96)	<0.01
Superior frontal	2.61 ± 0.15	2.55 ± 0.12	2.60 ± 0.13	1.24 (0.30)	0.03
Superior parietal	2.28 ± 0.11	2.27 ± 0.10	2.30 ± 0.10	0.82 (0.44)	0.02
Superior temporal	2.95 ± 0.20	2.90 ± 0.14	2.99 ± 0.16	2.06 (0.14)	0.06
Supramarginal	2.65 ± 0.10	2.62 ± 0.14	2.68 ± 0.11	1.47 (0.24)	0.04
Frontal pole	2.70 ± 0.30	2.64 ± 0.17	2.73 ± 0.26	0.91 (0.41)	0.03
Temporal pole	3.66 ± 0.37	3.63 ± 0.35	3.62 ± 0.35	0.07 (0.93)	<0.01

**Transverse temporal**	2.63 ± 0.23	**2.52 ± 0.18**	**2.68 ± 0.26**	**3.06 (0.05)**	**0.08**
Insula	3.12 ± 0.20	3.09 ± 0.16	3.17 ± 0.14	1.55 (0.22)	0.04

Abbreviations: ηp^2: partial eta squared

Notes: All analyses were covariated by smoking variable and marginal means are reported. Numbers in bold represent areas, comparing groups where statistically significant results were found.

.

Table S6.

Quantitative regional estimates for left hemisphere cortical thickness considering age of onset of gender incongruence

	Early onset GD n=12 mean ± SD	Late onset GD n=10 mean ± SD	Cisgender women n=28 mean ± SD	Cisgender men n=25 mean ± SD	F-Snedecor Statistic (p)	ηp^2
Bankssts	2.54 ± 0.17	2.61 ± 0.11	2.57 ± 0.17	2.59 ± 0.12	0.40 (0.75)	0.02
Caudal anterior cingulate	2.55 ± 0.16	2.72 ± 0.18	2.71 ± 0.20	2.71 ± 0.21	2.17 (0.10)	0.09
Caudal middle frontal	2.48 ± 0.12	2.63 ± 0.15	2.55 ± 0.12	2.55 ± 0.13	2.44 (0.07)	0.10
Cuneus	1.96 ± 0.15	2.03 ± 0.10	1.97 ± 0.12	2.01 ± 0.11	1.26 (0.30)	0.05
Entorhinal	3.49 ± 0.27	3.37 ± 0.36	3.34 ± 0.23	3.52 ± 0.30	2.10 (0.11)	0.08
Fusiform	2.84 ± 0.15	2.86 ± 0.10	2.84 ± 0.09	2.91 ± 0.08	2.38 (0.08)	0.09
Inferior parietal	2.49 ± 0.12	2.55 ± 0.12	2.52 ± 0.10	2.54 ± 0.11	0.71 (0.55)	0.03
Inferior temporal	2.87 ± 0.16	2.93 ± 0.16	2.85 ± 0.14	2.92 ± 0.14	1.16 (0.33)	0.05

Isthmus cingulate	2.43± 0.21	2.37 ± 0.18	2.45 ± 0.17	2.44 ± 0.14	0.53 (0.66)	0.02
Lateral occipital	2.29± 0.11	2.32 ± 0.14	2.27 ± 0.11	2.33 ± 0.10	1.21 (0.31)	0.05
Lateral orbitofrontal	2.64 ± 0.17	2.69 ± 0.10	2.64 ± 0.16	2.71 ± 0.13	1.14 (0.34)	0.05
Lingual	2.10 ± 0.12	2.11 ± 0.14	2.11 ± 0.11	2.17 ± 0.08	1.89 (0.14)	0.08
**Medial orbitofrontal**	2.44 ± 0.17	2.50 ± 0.11	**2.42 ± 0.11**	**2.53 ± 0.14**	**3.71 (0.02)**	**0.14**
Middle temporal	2.96 ± 0.17	2.99 ± 0.14	2.91 ± 0.11	2.99 ± 0.14	1.46 (0.23)	0.06
Parahippocampal	2.92 ± 0.28	2.84 ± 0.23	2.86 ± 0.28	2.95 ± 0.25	0.79 (0.51)	0.03
Paracentral	2.44 ± 0.13	2.53 ± 0.09	2.50 ± 0.13	2.51 ± 0.15	0.99 (0.40)	0.04
**Parsopercularis**	**2.59 ± 0.19**	2.70 ± 0.10	**2.62 ± 0.12**	**2.74 ± 0.11**	**5.10 (<0.01)**	**0.18**
Parsorbitalis	2.69 ± 0.25	2.74 ± 0.21	2.75 ± 0.19	2.73 ± 0.22	0.17 (0.92)	0.07
**Parstriangularis**	**2.46 ± 0.15**	**2.59 ± 0.14**	2.50 ± 0.10	2.57 ± 0.15	**3.25 (0.03)**	**0.12**
Pericalcarine	1.76 ± 0.13	1.74± 0.20	1.70 ± 0.14	1.72 ± 0.11	0.50 (0.68)	0.21

**Postcentral**	**2.09 ± 0.10**	**2.23 ± 0.06**	2.18 ± 0.11	**2.21 ± 0.10**	**4.53 (<0.01)**	**0.16**
Posterior cingulate	2.47 ± 0.17	2.53 ± 0.17	2.48 ± 0.14	2.53 ± 0.16	0.65 (0.58)	0.03
Precentral	2.59 ± 0.17	2.71 ± 0.14	2.69 ± 0.12	2.69 ± 0.12	2.32 (0.08)	0.09
Precuneus	2.45 ± 0.13	2.54 ± 0.07	2.49 ± 0.10	2.53 ± 0.11	2.02 (0.12)	0.08
Rostral anterior cingulate	2.94 ± 0.23	2.86 ± 0.14	2.87 ± 0.23	2.93 ± 0.20	0.71 (0.55)	0.03
Rostral middle frontal	2.33 ± 0.15	2.43 ± 0.11	2.35 ± 0.11	2.43 ± 0.13	2.83 (0.05)	0.11
Superior frontal	2.64 ± 0.19	2.78 ± 0.13	2.66 ± 0.13	2.73 ± 0.14	2.61 (0.06)	0.10
Superior parietal	2.23 ± 0.10	2.32 ± 0.10	2.28 ± 0.08	2.28 ± 0.11	1.58 (0.20)	0.06
Superior temporal	2.86± 0.21	3.00 ± 0.15	2.91 ± 0.13	2.97 ± 0.13	2.31 (0.08)	0.09
Supramarginal	2.59 ± 0.14	2.70 ± 0.12	2.63 ± 0.13	2.65 ± 0.13	1.53 (0.21)	0.06
Frontal pole	2.74 ± 0.25	2.79 ± 0.23	2.68 ± 0.22	2.78 ± 0.22	1.01 (0.39)	0.04
Temporal pole	3.58 ± 0.38	3.66 ± 0.33	3.51 ± 0.33	3.54 ± 0.40	0.39 (0.76)	0.02

Transverse temporal	2.49 ± 0.21	2.61 ± 0.22	2.56 ± 0.21	2.60 ± 0.25	0.78 (0.51)	0.03
Insula	2.96 ± 0.28	3.12 ± 0.16	3.02 ± 0.18	3.07 ± 0.16	1.61 (0.20)	0.07

Abbreviations: ηp^2: partial eta squared

Notes: All analyses were covariated by smoking variable and marginal means are reported. Numbers in bold represent areas, comparing groups where statistically significant results were found.

Table S7

Quantitative regional estimates for right hemisphere cortical thickness considering age of gender incongruence onset

	Early onset GD<br>n=10<br>mean ± SD	Late onset GD<br>n=12<br>mean ± SD	Cisgender women<br>n=28<br>mean ± SD	Cisgender men<br>n=25<br>mean ± SD	F-Snedecor Statistic<br>(p)	ηp^2
Bankssts	2.68 ± 0.21	2.75 ± 0.12	2.71 ± 0.14	2.73 ± 0.18	0.41 (0.74)	0.02
Caudal anterior cingulate	2.46 ± 0.17	2.62 ± 0.16	2.46 ± 0.17	2.52 ± 0.17	2.30 (0.08)	0.09
Caudal middle frontal	2.40 ± 0.18	2.55 ± 0.11	2.46 ± 0.11	2.44 ± 0.13	2.70 (0.05)	0.10
Cuneus	2.02 ± 0.11	2.07 ± 0.11	2.03 ± 0.13	2.09 ± 0.14	1.19 (0.32)	0.05
Entorhinal	3.61 ± 0.27	3.64 ± 0.28	3.49 ± 0.32	3.62 ± 0.29	1.12 (0.35)	0.05
**Fusiform**	2.87 ± 0.10	2.88 ± 0.12	**2.86 ± 0.09**	**2.94 ± 0.10**	**3.10 (0.03)**	**0.12**
Inferior parietal	2.56 ± 0.13	2.69 ± 0.10	2.60 ± 0.11	2.63 ± 0.10	2.60 (0.06)	0.10
Inferior temporal	2.85 ± 0.18	2.95 ± 0.16	2.85 ± 0.12	2.93 ± 0.13	2.33 (0.08)	0.09

Isthmus cingulate	2.52± 0.27	2.56 ± 0.23	2.44 ± 0.16	2.49 ± 0.18	1.07 (0.37)	0.04
Lateral occipital	2.32 ± 0.12	2.42 ± 0.13	2.37 ± 0.10	2.42 ± 0.11	2.46 (0.07)	0.10
Lateral orbitofrontal	2.56 ± 0.11	2.56 ± 0.09	2.59 ± 0.11	2.56 ± 0.12	0.32 (0.81)	0.01
Lingual	2.18 ± 0.14	2.15 ± 0.16	2.15 ± 0.10	2.21 ± 0.09	1.41 (0.25)	0.06
Medial orbitofrontal	2.38 ± 0.18	2.41 ± 0.12	2.38 ± 0.14	2.46 ± 0.14	1.90 (0.14)	0.08
Middle temporal	2.97 ± 0.19	3.03 ± 0.11	2.94 ± 0.14	3.04 ± 0.11	2.54 (0.06)	0.10
Parahippocampal	2.90 ± 0.23	2.91 ± 0.20	2.89 ± 0.21	2.95 ± 0.24	0.36 (0.79)	0.02
Paracentral	2.47 ± 0.14	2.60 ± 0.07	2.51 ± 0.12	2.53 ± 0.14	1.76 (0.16)	0.07
Parsopercularis	2.52 ± 0.20	2.61 ± 0.11	2.55 ± 0.15	2.60 ± 0.12	1.36 (0.26)	**0.06**
Parsorbitalis	2.61 ± 0.18	2.65 ± 0.16	2.68 ± 0.18	2.60 ± 0.17	1.08 (0.36)	0.04
Parstriangularis	2.41 ± 0.18	2.45 ± 0.18	2.40 ± 0.14	2.42 ± 0.16	0.25 (0.86)	0.01
Pericalcarine	1.76 ± 0.12	1.73 ± 0.20	1.70 ± 0.15	1.70 ± 0.14	0.51 (0.68)	0.02

**Postcentral**	**2.07 ± 0.09**	2.17 ± 0.09	**2.18 ± 0.12**	2.16 ± 0.11	**2.93 (0.04)**	**0.11**
Posterior cingulate	2.50 ± 0.13	2.58 ± 0.13	2.45 ± 0.15	2.50 ± 0.14	1.72 (0.17)	0.07
Precentral	2.54 ± 0.14	2.68 ± 0.12	2.58 ± 0.14	2.59 ± 0.12	2.36 (0.08)	0.09
Precuneus	2.49 ± 0.14	2.52 ± 0.10	2.50 ± 0.10	2.53 ± 0.10	0.51 (0.67)	0.02
Rostral anterior cingulate	2.84 ± 0.24	2.76 ± 0.16	2.81 ± 0.16	2.87 ± 0.16	1.04 (0.38)	0.04
Rostral middle frontal	2.20 ± 0.14	2.27 ± 0.13	2.22 ± 0.09	2.23 ± 0.10	0.89 (0.45)	0.04
Superior frontal	2.56 ± 0.18	2.66 ± 0.10	2.55 ± 0.12	2.60 ± 0.13	1.82 (0.15)	0.07
Superior parietal	2.24 ± 0.11	2.34 ± 0.07	2.27 ± 0.10	2.30 ± 0.10	2.52 (0.07)	0.10
**Superior temporal**	**2.88 ± 0.23**	**3.02 ± 0.15**	2.90 ± 0.14	2.99 ± 0.16	**2.87 (0.04)**	**0.11**
Supramarginal	2.61 ± 0.09	2.71 ± 0.10	2.62 ± 0.14	2.68 ± 0.11	2.26 (0.09)	0.09
Frontal pole	2.61 ± 0.25	2.81 ± 0.34	2.64 ± 0.17	2.73 ± 0.26	1.83 (0.15)	0.07
Temporal pole	3.61 ± 0.34	3.72 ± 0.41	3.63 ± 0.35	3.62 ± 0.35	0.20 (0.90)	<0.01

**Transverse temporal**	**2.52 ± 0.18**	**2.77 ± 0.20**	**2.52 ± 0.18**	2.68 ± 0.26	**4.86 (0.04)**	0.17
Insula	3.07 ± 0.20	3.19 ± 0.18	3.09 ± 0.16	3.17 ± 0.14	2.15 (0.10)	0.08

Abbreviations: ηp^2: partial eta squared

Notes: All analyses were covariated by smoking variable and marginal means are reported. Numbers in bold represent areas, comparing groups where statistically significant results were found.

Table S8

GO-Enrichment analysis of CBLL1 gene

Function	Type	Enrichment Score	Enrichment p-value
RNA N6-methyladenosine methyltransferase complex	cellular component	7.83	0.0004
mRNA editing complex	cellular component	7.83	0.0004
entry of bacterium into host cell	biological process	7.7	0.0004
mRNA methylation	biological process	7.14	0.0008
mRNA modification	biological process	6.7	0.0012
RNA methylation	biological process	5.5	0.004
entry into host	biological process	5.3	0.005
positive regulation of endocytosis	biological process	5.17	0.006
methyltransferase complex	cellular component	5.16	0.006
interaction with host	biological process	4.85	0.008

RNA modification	biological process	4.75	0.009
regulation of endocytosis	biological process	4.47	0.011
macromolecule methylation	biological process	4.37	0.013
negative regulation of cell adhesion	biological process	4.18	0.015
methylation	biological process	4.17	0.015
ubiquitin protein ligase activity	molecular function	4.16	0.015
ubiquitin ligase complex	cellular component	4.14	0.016
ubiquitin-like protein ligase activity	molecular function	4.13	0.016
nuclear speck	cellular component	3.84	0.021
ubiquitin-protein transferase activity	molecular function	3.77	0.023
ubiquitin-like protein transferase activity	molecular function	3.71	0.024
cell-cell adhesion	biological process	3.58	0.028

positive regulation of cell migration	biological process	3.58	0.028
positive regulation of cell motility	biological process	3.53	0.029
positive regulation of locomotion	biological process	3.5	0.03
positive regulation of cellular component movement	biological process	3.5	0.03
regulation of vesicle-mediated transport	biological process	3.5	0.03
mRNA metabolic process	biological process	3.36	0.034
protein ubiquitination	biological process	3.25	0.038
regulation of cell adhesion	biological process	3.24	0.039
transferase complex	cellular component	3.19	0.041
multicellular organism development	biological process	3.19	0.041
nuclear body	cellular component	3.16	0.042
protein modification by small protein conjugation	biological process	3.14	0.043

symbiotic process	biological process	3.14	0.043
regulation of cell migration	biological process	3.06	0.046
cell adhesion	biological process	2.99	0.05
